# Efficacy and safety of a restrictive blood transfusion protocol in gynecologic surgical patients

**DOI:** 10.1016/j.gore.2022.101059

**Published:** 2022-08-05

**Authors:** Rachel P. Mojdehbakhsh, Rana Al-Rubaye, Dandi S. Huang, Joseph Connor, Ahmed Al-Niaimi

**Affiliations:** a Department of Obstetrics and Gynecology, University of Wisconsin School of Medicine and Public Health, Madison, WI, United States; b University of Baghdad College of Medicine, Baghdad, Iraq; c Department of Pathology and Laboratory Medicine, University of Wisconsin School of Medicine and Public Health, Madison, WI, United States; d Division of Gynecologic Oncology, University of Wisconsin School of Medicine and Public Health, Madison, WI, United States

**Keywords:** Gynecology, Gynecologic oncology, Mortality, Postoperative complications, Restrictive blood transfusion

## Abstract

**Objectives:**

This study sought to compare differences in blood transfusion and surgical complication rates before and after the implementation of a restrictive blood transfusion protocol.

**Methods:**

On July 1, 2018, our institution implemented a restrictive blood transfusion protocol utilizing a hemoglobin trigger of less than 7 g/dL. Retrospective chart review was completed to review patients undergoing major abdominal surgery by the gynecology and gynecologic oncology services 18 months before, and after initiation of the transfusion protocol. Outcomes included number of patients, units transfused and postoperative complication rates. Complications included reoperation on the day of admission, surgical site infections, wound disruptions, pulmonary, renal, central nervous system, and cardiovascular complications, as well as deep venous thromboses, readmissions, and 30-day mortality.

**Results:**

There were 290 people in the pre- and 449 patients in the post-protocol group. A similar number of patients received blood transfusions in both groups (9.3% versus 10.6% p = 0.57). However, significantly fewer units of blood were given post-protocol initiation. For every patient who received a transfusion pre-protocol, 2.66 units were administered compared to 1.2 units after the protocol was initiated (p = 0.003). All postoperative complications were not significantly different between groups (p > 0.05). Individual postoperative complications were combined and analyzed using a clustered approach to detect rates of complications more conservatively. Both the 7-system (5.1% versus 4.9%, p = 0.90) and 8-system (5.5% versus 4.9%, p = 0.72) clustered analyses were not significantly different before and after the initiation of the transfusion protocol.

**Conclusions:**

A restrictive transfusion protocol is effective in decreasing the number of units of blood transfused without affecting postoperative complication rates in gynecologic surgery patients.

## Highlights

• Restrictive blood transfusion protocol decreased number of blood transfusions given to gynecologic surgical patients.
• Restrictive blood transfusion protocol did not change postoperative complication rates in gynecologic surgical patients.
• Restrictive blood transfusion protocols are shown to be safe and effective in a gynecologic surgical population.

## Graphical abstract

**Figure:**
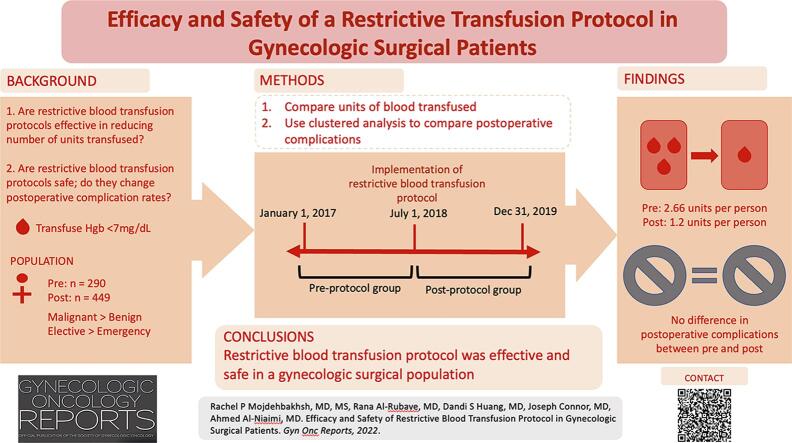

## 1 Introduction

Major abdominal surgeries for gynecologic indications, both benign and malignant, are often for the purposes of abnormal uterine bleeding or in patients with existing anemia. These surgeries often can incur excessive operative blood loss. Blood transfusion efficacy and safety are relevant and understudied in these populations.

In 2016, the American Association of Blood Banks (AABB) recommended a hemoglobin cutoff of 7 g/dl for transfusion of red blood cells in hemodynamically stable and critically ill patients. A threshold of 8 g/dl is recommended for patients undergoing cardiac or orthopedic surgery or those with cardiovascular disease (Carson et al., 2016). Blood transfusions are not benign interventions and carry the risks of infection, hemolytic reactions, immunosuppression, transfusion-related acute lung injury, and alloimmunization. Transfusion related immune modulation is a process that has been demonstrated in various patients with solid tumors, and is thought to be associated with duration of storage of blood products, though it is poorly understood (Al-Refaie et al., 2012, Harlaar et al., 2012). Additional research has shown increased tumor recurrence rates and nosocomial infection rates in patients receiving blood transfusions thought to be associated with iatrogenic immunosuppression (Theodoraki et al., 2014).

Evidence to support restrictive blood transfusion protocols varies depending on the study population. With respect to non-surgical patients, the Transfusion Requirements in Critical Care randomized control trial specifically enrolled critically ill patients and demonstrated 30-day mortality was similar in patients receiving a liberal versus restrictive transfusion protocol. Moreover, 30-day mortality was significantly lower in patients receiving the restrictive protocol in the less-critically ill study population (Hebert et al., 1999). Various studies looking at patients with cardiac comorbidities who are undergoing major surgery suggest a restrictive transfusion protocol may be harmful in these cases (Hovaguimian and Myles, Jul. 2016, Carson et al., 2015). Data regarding orthopedic surgical patients and restrictive transfusion is mixed and in part seems to be dependent on the comorbidities of the population (Martin et al., 2017).

In non-cardiac surgical patients broadly, restrictive protocols have been shown either not affect or decrease perioperative complications and postoperative morbidity (Martin et al., 2017). In a large study of surgical oncology patients, multivariate analyses revealed intraoperative transfusions negatively impacted postoperative outcomes (Al-Refaie et al., 2012). With respect to gynecologic oncology specifically, existing evidence suggests that use of restrictive blood transfusion protocols may decrease surgical site infection or have no affect on postoperative morbidity (Mark et al., 2019, Prescott et al., 2019, Boone et al., Jan. 2014).

Our primary aim was to evaluate the efficacy of a restrictive blood transfusion protocol initiated at a large academic hospital by comparing blood transfusion data pre- and post-initiation of our institutional restrictive blood transfusion protocol. Secondarily, we aimed to compare postoperative outcomes before and after protocol initiation in a gynecologic surgical population. Uniquely, our postoperative complication analysis included a clustered analysis to detect differences more robustly in complication rates in this population by combining surgical site infection, wound disruptions, pulmonary, renal, cardiovascular, and CNS complications, DVT rates and 30-day mortality into one outcome. We hypothesized that implementing a restrictive blood transfusion protocol in a gynecologic patient population was both effective at reducing the number of units of blood given and safe for this patient population.

## 2 Materials and methods

This study was designed as a quality improvement effort using a quasi-experimental design. We conducted a retrospective chart review of the Gynecologic Oncology Longitudinal Data Collection and Utilization Project (GOLD-CUP) institutional database and cross referenced this with the ACS National Surgery Quality Improvement Program (NSQIP) to review patients undergoing major abdominal surgery by the gynecology and gynecologic oncology services. Inclusion criteria included all major abdominal surgeries performed by the gynecologic and gynecologic oncology services for gynecologic indications from January 1, 2017, to December 31, 2019. Patients were excluded if they had no longitudinal data for chart review.

On July 1, 2018, our institution implemented a restrictive blood transfusion protocol based on the AABB guidelines, recommending against blood transfusion in hemodynamically stable patients when hemoglobin is above 7 g/dL, unless the indication was cardiovascular (Blood Products Transfusion: Indications-Adult-Inpatient/Ambulatory/Emergency Department-Clinical Practice Guideline, 2021). In our pre-protocol group, we included patients who underwent surgery from January 1, 2017, to June 30, 2018, and compared these to patients in the post-protocol group who underwent surgery between July 1, 2018, to December 31, 2019.

Thirty-day postoperative outcomes were collected. Outcomes for the transfusion data included number of patients who received a blood transfusion and number of units given. Postoperative complication data included reoperation on the day of admission, surgical site infections, wound disruptions, pulmonary, renal, central nervous system (CNS), and cardiovascular complications, as well as deep venous thromboses (DVT), readmissions, and 30-day mortality. Demographic data were also collected. Surgical data including wound class, route of surgery, pathology, and emergent status were also collected. The 7-system clustered analysis combined surgical site infection, wound disruptions, pulmonary, renal, cardiovascular, and CNS complications in addition to DVT rates. The 8-system clustered model combined the same complications as the 7-system model in addition to 30-day mortality.

Transfusion and postoperative complication data were then analyzed using the statistical program Libxlsxwriter (Version 1.9.48 (277)). Categorical variables were analyzed using chi-squared and Fisher’s exact tests. Continuous variables were analyzed using student t-tests. A clustered analysis was also completed to further examine the significance of surgical complications (Erekson et al., 2011, Meguid et al., 2016, Meguid et al., 2016). A *p*-value of < 0.05 was considered statistically significant. This study was deemed exempt by the Institutional Review Board.

## 3 Results

A total of 739 patients were included. There were 290 people in the pre-protocol group and 449 patients in the post-protocol group. The mean age of patients in each group were not different: 57.6 years old in the pre-protocol group, 59.2 years old in the post-protocol group. Body mass index (BMI) was the only significantly different characteristic between the pre- and post-protocol groups with the post-protocol group having a higher BMI of 33.1 compared to 31.8. All other demographic and clinical characteristics were not different between the groups, including medical comorbidities. With respect to surgical characteristics, significantly more patients underwent surgery for oncologic indications compared to benign indications in both the pre- and post-protocol group (56.2% vs. 72%, p < 0.0001). Additionally, there were significantly more elective than emergency surgeries both before and after the initiation of the restrictive transfusion protocol (p < 0.0001) (Table 1).

**Table 1:** Descriptive characteristics of pre-protocol and post-protocol group.

Group				
		Pre-Protocol	Post-Protocol	
		1/1/2017 – 6/30/2018	1/7/2018 – 12/31/2019	
		n = 290	n = 449	
Demographics				p value
Age	Mean (SD)	57.6 (14.4)	59.2 (13.2)	0.12
Race/Ethnicity				
	Non-Hispanic-White	267 (92.1%)	416 (92.7%)	
	Non-Hispanic-Black	10 (3.4%)	23 (5.1%)	
	Asian/Pacific Islander	5 (1.7%)	4 (0.9%)	
	Native American	4 (1.4%)	2 (0.4%)	
	Unknown	4 (1.4%)	4 (0.9%)	
	Hispanic	7 (2.4%)	15 (3.3%)	
BMI	Mean	31.8	33.1	<0.001
Comorbidities	Diabetes	33 (11.4%)	66 (14.7%)	0.20
	Hypertension	118 (40.6%)	175 (38.9%)	0.64
	Heart Failure	1 (0.3%)	1 (0.2%)	0.75
	Tobacco Use	37 (12.7%)	36 (8.0%)	0.04
	COPD	3 (1%)	6 (1.3%)	0.71
	Steroid Use	14 (4.8%)	14 (3.1%)	0.24
	ASA			0.06
	1	15 (5.2%)	4 (0.9%)	
	2	180 (62.1%)	247 (55.0%)	
	3	93 (32.1%)	193 (43.0%)	
	4	2 (0.7%)	5 (1.1%)	
ECOG Functional Status				
	0–2	286 (98.6%)	447 (99.5%)	0.19
	3–4	4 (1.3%)	2 (0.4%)	
Transfusion prior to surgery		1 (0.3%)	1 (0.2%)	0.75
Hematocrit prior to surgery	Mean	37.9	38.6	0.29

When blood transfusion data were analyzed, a similar number of patients received blood transfusions in both groups (9.3% vs. 10.6% p = 0.57). However, significantly fewer units of blood were given post-protocol initiation. For every patient who received a transfusion pre-protocol, 2.66 units were administered compared to 1.2 units after the protocol was initiated (p = 0.003) (Table 1).

All postoperative complications were not significantly different between groups: reoperation on day of admission (p = 0.38), surgical site infection (p = 0.54), wound disruption (p = 0.82), pneumonia (p = 0.08), reintubation (p = 0.22), pulmonary embolism (p = 0.99), renal insufficiency (0.08), delirium (p = 0.22), coronary artery disease (p = 0.42), DVT (p = 0.25), 30-day mortality (p = 0.22), readmissions (p = 0.37). Clustered analyses were performed to interpret postoperative complications that occur at low rates. Both the 7-system (5.1% vs. 4.9%, p = 0.90) and 8-system (5.5% vs. 4.9%, p = 0.72) clustered analyses were not significantly different before and after the initiation of the transfusion protocol (Table 2).

**Table 2:** Surgical and Transfusion Data.

Groups				
		Pre-Protocol	Post-Protocol	
		1/1/2017 – 6/30/2018	1/7/2018 – 12/31/2019	
		n = 290	n = 449	p value
Surgical Status				<0.0001
	Elective	274 (91.5%)	445 (99.1%)	
	Emergency	16 (8.5%)	4 (0.9%)	
Surgical Approach				0.123
	Laparoscopy	163 (56.2%)	278 (61.9%)	
	Laparotomy	127 (43.8%)	171 (38.1%)	
Service				<0.0001
	Gynecologic Oncology	199 (68.5%)	395 (87.9%)	
	Gynecology	91 (31.5%)	54 (12.1%)	
Pathology				
Malignant		163 (56.2%)	323 (72%)	<0.0001
	Uterine cancer	98 (60%)	214 (66%)	
	Ovarian cancer	50 (31%)	90 (28%)	
	Cervix cancer	15 (9%)	19 (6%)	
Benign		127 (43.7%)	126 (28%)	
Transfusion Data				
Patients received transfusion		27 (9.3%)	48 (10.6%)	0.567
Units of blood given		72	52	0.0031
Postoperative complications				
Reoperation POD0		3 (1%)	8 (1.8%)	0.381
Infection				
	SSI (total)	7 (2.4%)	14 (3.1%)	0.535
	Superficial	5 (71.4%)	11 (78.6%)	
	Deep	0	1 (7.1%)	
	Organ space	2 (28.6%)	2 (1.4%)	
	UTI	11 (2.8%)	11 (2.5%)	0.803
	C. Diff	0	1 (0.2%)	0.424
	Sepsis	2 (0.3%)	2 (0.5%)	0.470
Wound Disruption		1 (0.3%)	2 (0.5%)	0.818
Pulmonary				
	Pneumonia	2 (0.68%)	0	0.08
	Reintubation	1 (0.34%)	0	0.216
	PE	2 (0.68%)	3 (0.67%)	0.987
	Vent > 48 hr.	0	0	
Renal				
	Renal insufficiency	2 (0.68%)	0	0.08
Central Nervous System				
	Cerebrovascular accident	0	0	
	Delirium	1 (0.34%)	0	0.216
Coronary Artery Disease		0	1 (0.2%)	0.424
Deep Vein Thrombosis		0	2 (0.5%)	0.253
30-Day Mortality		1 (0.3%)	0	0.216
Readmission		6 (2%)	14 (3.1%)	0.365
Clustering of post-operative complications (The 7 most relevant complications outcome) (7 systems are: SSI, Wound, Respiratory, CVS, CNS, Renal and DVT-PE)				
		15 (5.1%)	22 (4.9%)	0.902
Clustering of post-operative complications (The 8 most relevant complications outcome) (8 systems are: SSI, Wound, Respiratory, CVS, CNS, Renal, DVT-PE, and mortality)				
		16 (5.5%)	22 (4.9%)	0.718

## 4 Discussion

This study found that the implementation of a restrictive blood transfusion protocol is effective in reducing the number of units of blood transfused. Moreover, we demonstrated that postoperative complications do not differ in a gynecologic surgical population after the initiation of this protocol. These findings support that adoption of restrictive blood transfusion protocols are not harmful to patients in this population.

Restrictive blood transfusion protocols have been studied in cardiac, vascular, orthopedic, and oncologic surgical patients, or those who are critically ill or have cardiovascular disease. In gynecologic oncologic patients undergoing surgery, one study of 582 patients showed no difference in postoperative infections, thrombotic events, or mortality between patients who received restrictive blood transfusions (Boone et al., 2014). A different study of a gynecologic oncology population showed a reduction in superficial and deep surgical site infections after the implementation of a restrictive transfusion protocol, though the authors comment on the concomitant implementation of a surgical site infection task force introduced during that time (Mark et al., 2019). In a study similarly modeled to this one that compared the administration of blood products before and after the initiation of a restrictive blood transfusion protocol as well as postoperative adverse events, Prescott et al. demonstrated a decrease in surgical site infection and no difference in 30-day mortality, VTE or cardiac rates between groups (Prescott et al., 2019). Our data expands on these findings to include analysis of reoperation on day of admission, reintubation, renal insufficiency, delirium, and readmissions. Additionally, by clustering these postoperative complications and again showing no difference between the pre- and post-protocol groups, we have more robustly demonstrated the safety of a restrictive blood transfusion protocol in a gynecologic surgical population.

In this study, we included both benign and oncologic patients undergoing major abdominal surgery for gynecologic indications. We found no difference in postoperative complications including infection, thrombotic disease, or mortality. These results were further bolstered by clustering complications as one outcome to account for low incidence of these complications. These data provide more evidence to support the use of restrictive blood transfusion protocol in a gynecologic patient population in the existing landscape of growing evidence.

One limitation of this study was the inclusion of both benign and malignant indications for gynecologic surgery, which could confound the rates of postoperative complications, as there was a larger proportion of benign indications in the pre-protocol group. Additionally, both elective and emergent surgeries were included, though the vast majority were elective. Indications for blood transfusions and resulting complications in an emergent population could affect our overall comparison. Another limitation of this study involves the lack of data detailing timing of blood transfusion, estimated blood loss and reason for transfusion as well as preoperative optimization with blood transfusions or iron. This is in part because gynecologic patient data was abstracted from a larger surgical dataset initially collected by the institutional blood bank, and therefore did not encompass all variables pertinent and of interest to gynecology in particular.

To our knowledge, there is no data regarding the use of restrictive transfusion protocols in strictly a benign gynecologic surgical population. Additional research is needed to parse out any differences in postoperative complications or mortality in this population, as chronic or severe acute anemia is often an indication for major gynecologic surgery in this population. More longitudinal data could be beneficial to elucidate long term outcomes and complications in specifically an oncologic population. The effects of immune modulation after transfusion are not well understood with respect to longevity or potential interaction with subsequent immunosuppressive therapies or surgeries.

Our results demonstrated that a restrictive blood transfusion protocol was successful at significantly decreasing units of blood transfused based on evidence-based guidelines from the AABB. Furthermore, our results showed that adopting a restrictive blood transfusion protocol did not increase perioperative complications and is safe in a gynecologic surgical population. Based on our results, we plan to continue to adhere to and promote a restrictive blood transfusion protocol in this patient population.

As a quality improvement effort, this study was able to effectively compare transfusion, surgical, and postoperative data before and after the initiation of a restrictive blood transfusion protocol in a gynecologic patient population. We were able to obtain data for this patient population with the use of an institutional quality improvement database cross referenced with NSQIP for accuracy. This process provided for a robust representation of our patient population leading to clinically applicable results. Moreover, the inclusion of a clustered postoperative complication analysis has, to our knowledge, not been implemented in comparable studies and further reinforces our conclusions and adds strength to the existing body of evidence at large.

## 5 Conclusions

We conclude that at an institutional level, the implementation of a restrictive blood transfusion protocol is not only effective in decreasing blood product utilization but also safe as demonstrated by no increase in postoperative complication rates in a gynecologic surgical population.

### CRediT authorship contribution statement

**Rachel P. Mojdehbakhsh:** Writing – original draft. **Rana Al-Rubaye:** Writing – review & editing. **Dandi S. Huang:** Writing – review & editing. **Joseph Connor:** Conceptualization, Investigation, Writing – review & editing. **Ahmed Al-Niaimi:** Conceptualization, Methodology, Writing – review & editing, Supervision, Project administration.

## Declaration of Competing Interest

The authors declare that they have no known competing financial interests or personal relationships that could have appeared to influence the work reported in this paper.
